# A novel rhodamine B fluorescence probe for rapid identification of different amino acids by high efficiency fluorescence spectrum-mass spectrometry

**DOI:** 10.3389/fchem.2024.1409420

**Published:** 2024-09-30

**Authors:** Xiujie Duan, Tao Jin, Boneng Mao, Shihe Shao, Lei Zhao

**Affiliations:** ^1^ Department of Clinical Laboratory, Affiliated Hospital of Jiangsu University, Zhenjiang, China; ^2^ Department of Gastroenterology, Yixing Hospital Affiliated to Jiangsu University, Yixing, China; ^3^ School of Pharmaceutical Sciences, Changchun University of Chinese Medicine, Changchun, China

**Keywords:** rhodamine B, rapid identification, different amino acid, fluorescence spectrum, mass spectrometry

## Abstract

**Introduction:**

Rapid detection of amino acids plays an important role in the field of medical diagnosis. By combining Rhodamine B with triphenylamine, a novel double-response fluorescence probe (E)-4-((4-(((3′,6′-bis(diethylamino)-3-oxospiro[isoindoline-1,9′-xanthen]-2-yl)imino)methyl)phenyl)(phenyl)amino)benzaldehyde (RBTPA) was prepared for rapid identification of different amino acids.

**Methods:**

Under daylight and 365 nm irradiation, it was found that the color change was most bright at pH = 3, and changed to dim at pH = 4. When pH = 3 and pH = 4, the photophysical properties of the two strong acids are very different. The maximum redshift of UV absorption light is 110 nm, and the maximum fluorescence emission intensity is 4 times different.

**Results and Discussion:**

In order to further observe their binding structure analysis with different amino acids, qualitative analysis of each response structure was determined by mass spectrometry according to different molecular weights. The fluorescence probe RBTPA has two different isomers for recognition response in aldehyde group and imine group, respectively.

## Graphical Abstract

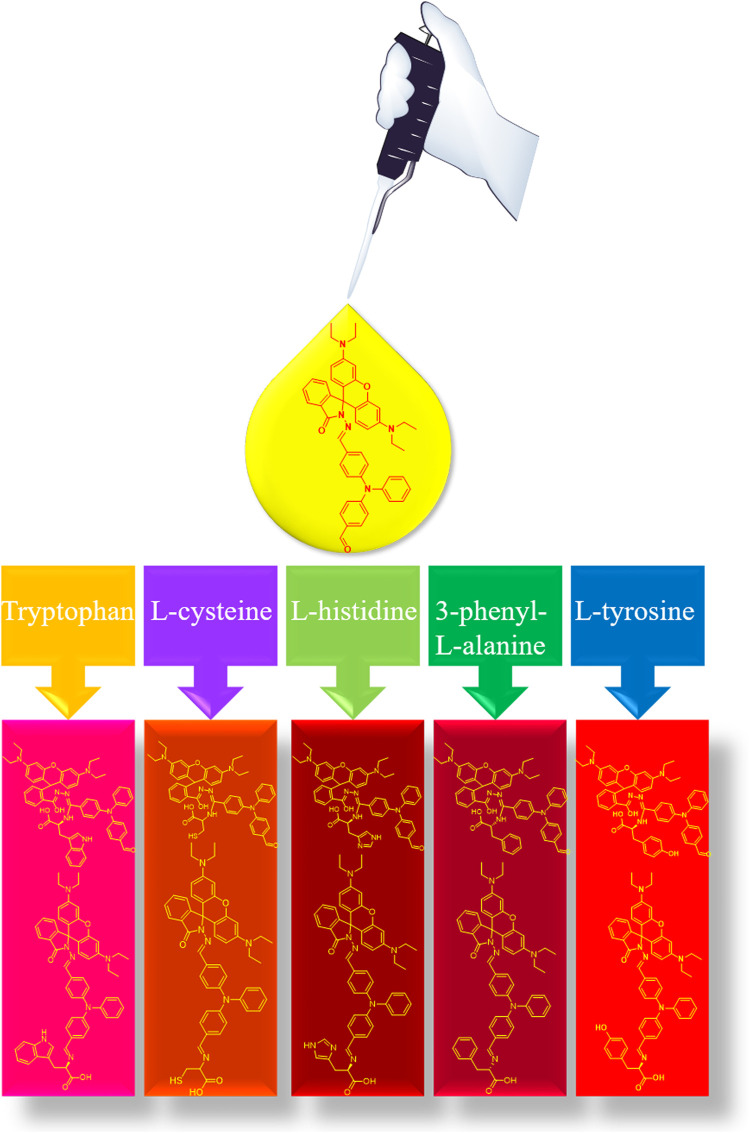

## Introduction

Amino acids are the “raw materials” that the human body uses to construct different proteins. These proteins are used to build or repair cell tissues, make hormones, enzymes, antibody proteins, and other substances, as well as to control metabolism and immunity (Bi et al., 2024; Ye et al., 2024). The foundation of human health is an adequate and balanced amount of amino acids (Guo et al., 2024; Li et al., 2024). People will be in a sub-health state and more susceptible to illness if there is no supply of amino acids, since this will impair their immune systems and other regular processes (Jin et al., 2020a; Jin et al., 2020b).

Currently, an imbalance in amino acids is responsible for about 300 different types of illnesses, including immune system disorders, cancers, viral diseases, nervous system disorders, and geriatric diseases (Yin et al., 2024; Lu et al., 2024). The body’s imbalance of amino acids can give information about a wide range of linked disorders as well as a thorough and dynamic evaluation of the levels of neurotransmitter metabolism, urea cycle, and amino acid functional metabolism. Additionally, early risk assessment may be carried out for malignant and chronic illnesses (Xiao et al., 2020; Deng et al., 2024; Fan et al., 2024). At present, major medical institutions mainly detect small molecule substances such as amino acids through full spectrum amino acid detection LC-MS/MS technology and small molecule fluorescent probe used in laboratories (Deng et al., 2021; Wang et al., 2021; Zhao et al., 2024; Shao et al., 2022; Schumann et al., 2023). Such as formaldehyde titration method: this method is simple to operate and has high accuracy, but it is prone to interference by other acidic substances. High performance liquid chromatography: This method has good separation effect, high sensitivity, and can detect a variety of amino acids at the same time. However, the operation is complicated, the cost is high, and the use of organic solvents is required. Although they have high sensitivity and fast detection speed, their equipment requirements are high, the fluorescence effect is poor, and the rapid and immediate detection of different amino acids cannot be visually monitored (Ye et al., 2024; Hu et al., 2022; Hao et al., 2024). Therefore, how to develop an efficient, qualitative and rapid visualization fluorescent probe to solve this difficulty has important value and significance (Rong et al., 2024).

In this paper, we designed a new type of double response binding site, using rhodamine B’s amide and triphenylamino aldehyde groups to combine with different amino acids or sulfhydryl groups to emit strong fluorescence chromophore, to achieve simple, qualitative and visual fluorescence effect.

## Experimental section

### Reagents and instrumentation

All reagents were purchased commercially and further purified when used. All the reactions involved were monitored by thin-layer chromatography (TLC) and analyzed with ultraviolet lamps at 254 and 365 nm. Products were separated and purified by column chromatography (200–300 mesh silica gel). NMR were recorded by Bruker avance II instrument in deuterium chloroform (400 MHz for ^1^H and 100 MHz for ^13^C). Chemical shifts are reported in ppm, versus internal tetramethylsilane (TMS) as a standard. The mass spectrum was obtained on a Thermo LXQ by liquid chromatgraphy-ion trap mass spectrometry. Absorption spectra were recorded with a UV-2550 spectrophotometer. The fluorescence emission spectra were measured by Shimazu RF-5301PCS fluorescence spectrophotometer with excitation wavelength of 500 nm. The molecular weight by MS-ESI (Jeol LTD JMS-HX 110/110A) was performed.

### Synthesis

#### (E)-4-((4-(((3′,6′-bis(diethylamino)-3-oxospiro[isoindoline-1,9′-xanthen]-2-yl)imino)methyl) phenyl)(phenyl)amino)benzaldehyde

2-amino-3′,6′-bis(diethylamino)spiro[isoindoline-1,9′-xanthen]-3-one (0.58 g, 1.3 mmol) was dissolved in ethanol (3 mL), 4,4'-(phenylazanediyl)dibenzaldehyde (0.38 g, 1.3 mmol) and acetic acid (0.1 mL) were added. The reaction was stopped after magnetic stirring at room temperature for 3 h. Yellowish compound (0.52 g, 56%) was isolated by column chromatography (petroleum ether: EtOAc = 4:1). ^1^H-NMR: (400 MHz, CDCl_3_, ppm): 8.65 (s, 1H), 7.98–7.97 (dd, *J* = 3.0 Hz, 1H), 7.48–7.39 (m, 1H), 7.23–7.19 (m, 1H), 7.12–7.10 (m, 1H), 7.03–6.98 (m, 1H), 6.91–6.89 (d, *J* = 6.0 Hz, 2H), 6.53–6.51 (d, *J* = 6.0 Hz, 2H), 6.42 (s, 2H), 6.26–6.23 (dd, *J* = 3.0 Hz, 2H), 4.15–4.10 (m, 1H), 3.34–3.29 (m, 9H), 2.05 (s, 2H), 1.28–1.24 (t, *J* = 6.0 Hz, 1H), 1.17–1.13 (t, *J* = 6.0 Hz, 12H); ^13^C NMR (100 MHz, CDCl_3_): δ (ppm)164.73, 153.21, 151.70, 148.89, 148.55, 129.43, 128.53, 128.44, 125.49, 123.83, 123.08, 107.29, 97.44, 66.06, 60.40, 44.33, 21.07, 14.23, 12.67. ITMS (ESI) calcd for C_29_H_33_BF_2_N_6_O_2_S [M + H]^+^ m/z 740.92; found 739.85.

### Spectroscopic properties

Ultraviolet-visible (UV-vis) spectra were recorded using a 1 cm quartz tube on a UV-2550 spectrophotometer, and fluorescence spectra were performed at room temperature on a Shimadzu RF-5301PCS fluorescence spectrophotometer. The making phosphate buffers with pH values of 3.0, and 4.0 each buffer was mixed with different equivalents of amino acids. Appropriate amount of RBTPA to be measured was weighed and dissolved in EtOH to prepare a solution with a concentration of 20 μg/mL for use. Different concentrations of amino acids in solvents were detected by ultraviolet and fluorescence methods. The wavelength range of UV-Vis is 450–700 nm. The fluorescence of the compound was obtained at the optical path of 10 mm and the excitation wavelength of 560 nm, and the emission wavelength was recorded in the range of 400–700 nm.

### Fluorescence stability data

The fluorescence stability data was determined using a Japanese Shimadzu RF-5301PC fluorescence spectrophotometer, DMSO was used as the solvent, and the sample concentration was continuously diluted with DMSO until the fluorescence intensity of the RBTPA remained stable. Fluorescence intensity stability over 5 min and 10 min was measured, respectively.

### Mass spectrometry

The mass spectrums were obtained at the Thermo LXQ by liquid chromatgraphy-ion trap mass spectrometry. To verify this mechanism, a random selection of RBTPA solutions containing 1.0 mg/mL of different amino acid solutions was performed by mass spectrometry to confirm our proposed mechanism.

## Result and discussion

### Design

**SCHEME 1:**
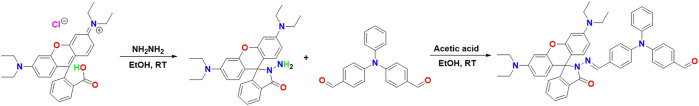
Protocol of RBTPA.

The design concept of the amino acid fluorescence probe in this paper is to consider the double response effect. Triphenylamino aldehyde, the most widely used luminescent electron donor in the semiconductor field, is selected as the terminal binding point, and the fluorophore rhodamine B is introduced. This design not only greatly reduces the fluorescence quenching problem of the probe, but also has the rapid response function in the acid environment in the overall structure, the best of both worlds (Scheme 1).

**FIGURE 1:**
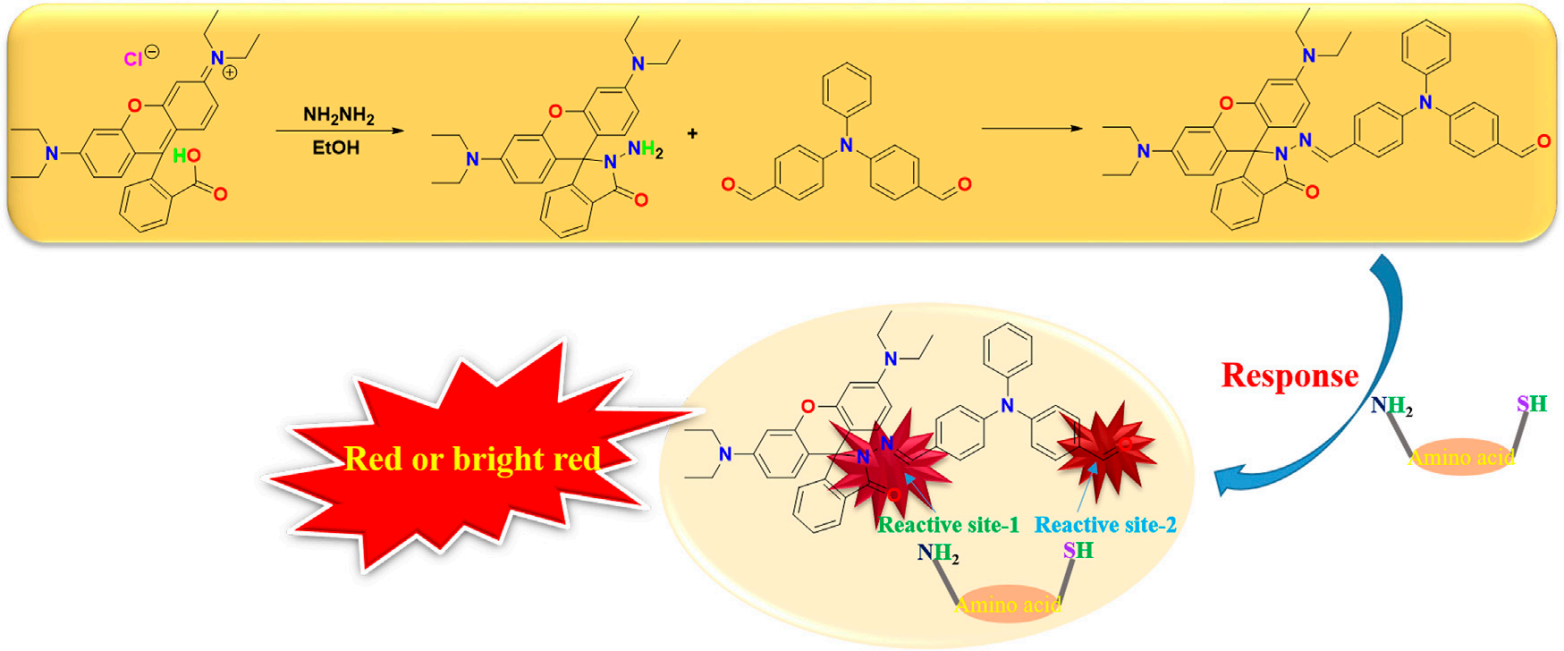
Reaction mechanism of different amino acids.

The rhodamine B structure’s iminopentane nitrogen heterocyclic ring is easily opened, and the pentane nitrogen heterocyclic ring is extremely unstable, forming an open ring rhodamine B structure and releasing strong fluorescence when the external electron is combined with imine through the Michael addition reaction. Furthermore, the conjugation of the big link is enhanced and given a high fluorescence effect and stability when triphenylamino aldehyde is combined with amino acids. Due to the change of the overall structure, different amino acid compounds produce different color changes under the irradiation of 365 nm wavelength, and the qualitative analysis of their mass spectrometry was determined by LC-MS, so as to detect the identification of different amino acids (Figure 1).

**FIGURE 2:**
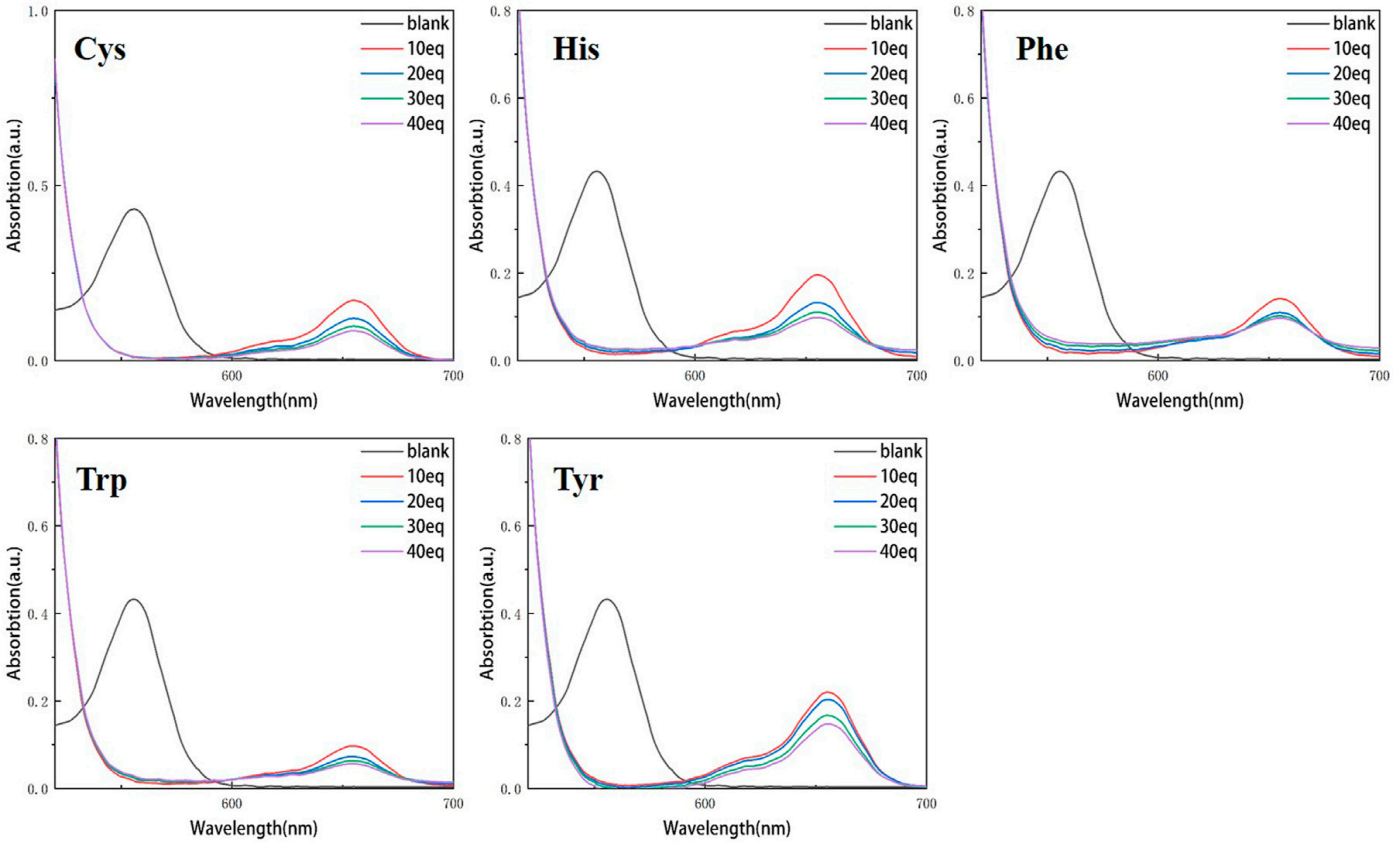
UV spectrum of RBTPA probe at different concentrations and different amino acids (pH = 3).

**FIGURE 3:**
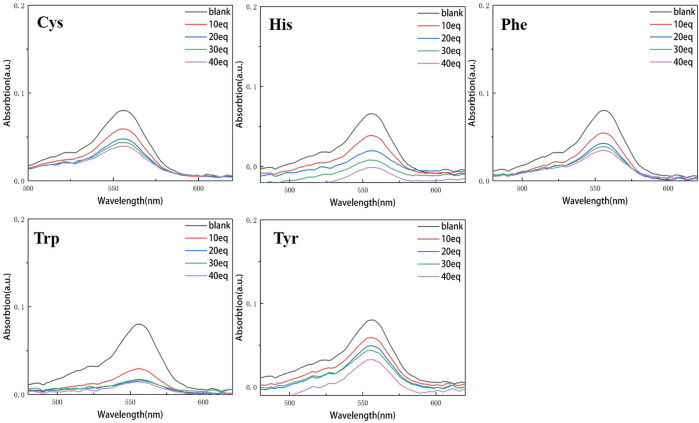
UV spectrum of RBTPA probe at different concentrations and different amino acids (pH = 4).

Continuous testing from 0 to 40 equivalents were conducted in order to examine the variations of RBTPA with different equivalents and different amino acids at pH = 3 and 4 in ethanol solvents. As can be seen in Figure 2, RBTPA’s blank absorption wavelength is 562 nm at pH = 3. The absorption strength of RBTPA is marginally decreased when mixed with other amino acids, yet it exhibits a clear wavelength redshift of 665 nm, with a gap of approximately 100 nm between the blank and control groups. The blank group and the control group showed no change in absorption wavelength at pH = 4, but there was a considerable drop in absorption intensity to around 0.8, with the total difference remaining at the same level. The outcomes demonstrated that the fluorescence impact and amino acid double response binding capacity improved with increasing acidity of RBTPA. Conversely, at high pH values, the reaction is less and fluorescence quenching occurs (Figures 2, 3) (Table 1).

**TABLE 1:** UV absorption properties of RBTPA in different amino acids.

UV absorption (pH)	Blank	Different amino acids				
λ_abs max_, pH = 3	562 nm	Cys	His	Phe	Trp	Try
		665 nm	667 nm	664 nm	665 nm	667 nm
λ_abs max_, pH = 4	560 nm	Cys	His	Phe	Trp	Try
		562 nm	563 nm	560 nm	561 nm	560 nm

**FIGURE 4:**
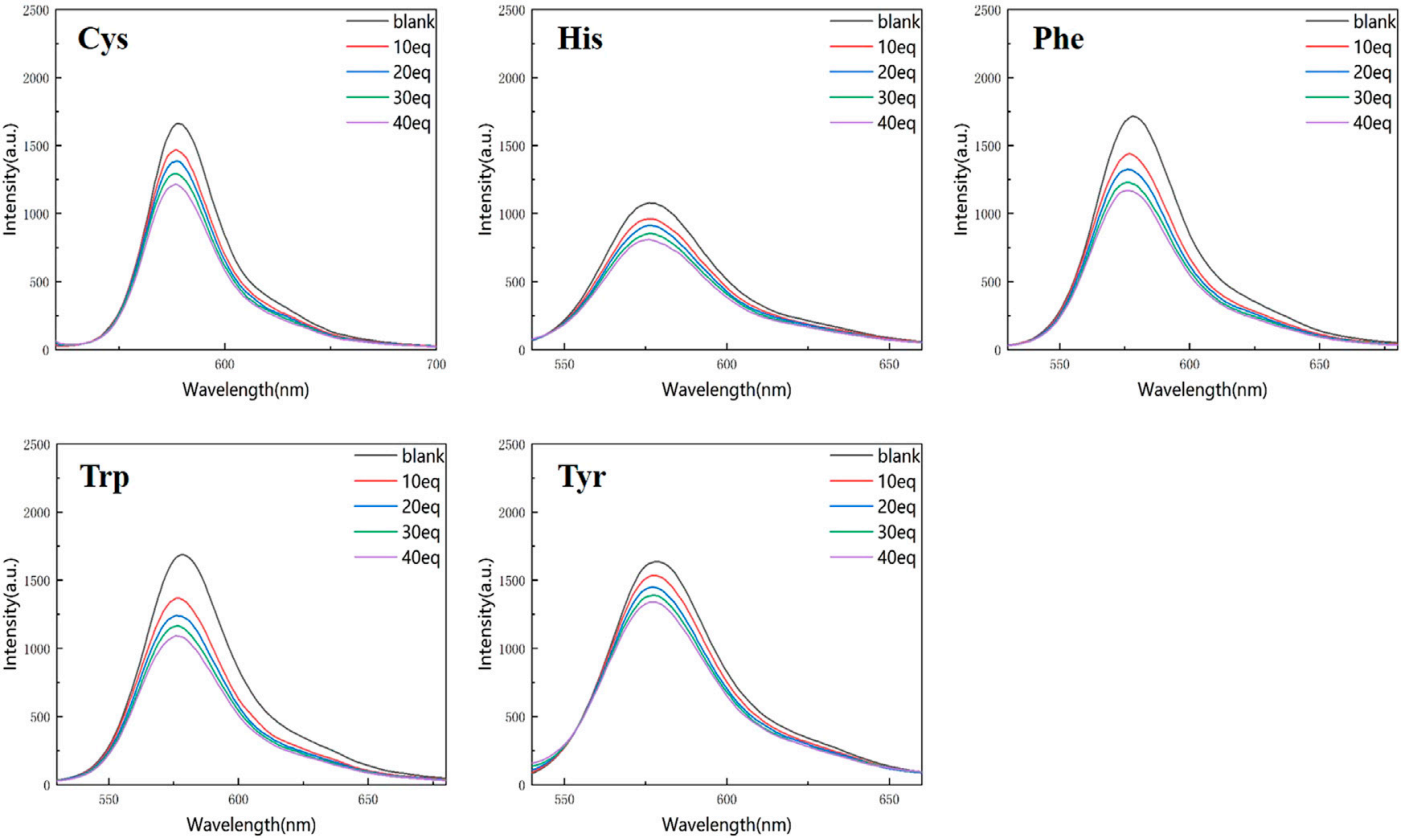
Fluorescence spectrum of RBTPA probe at different concentrations and different amino acids (pH = 3).

**FIGURE 5:**
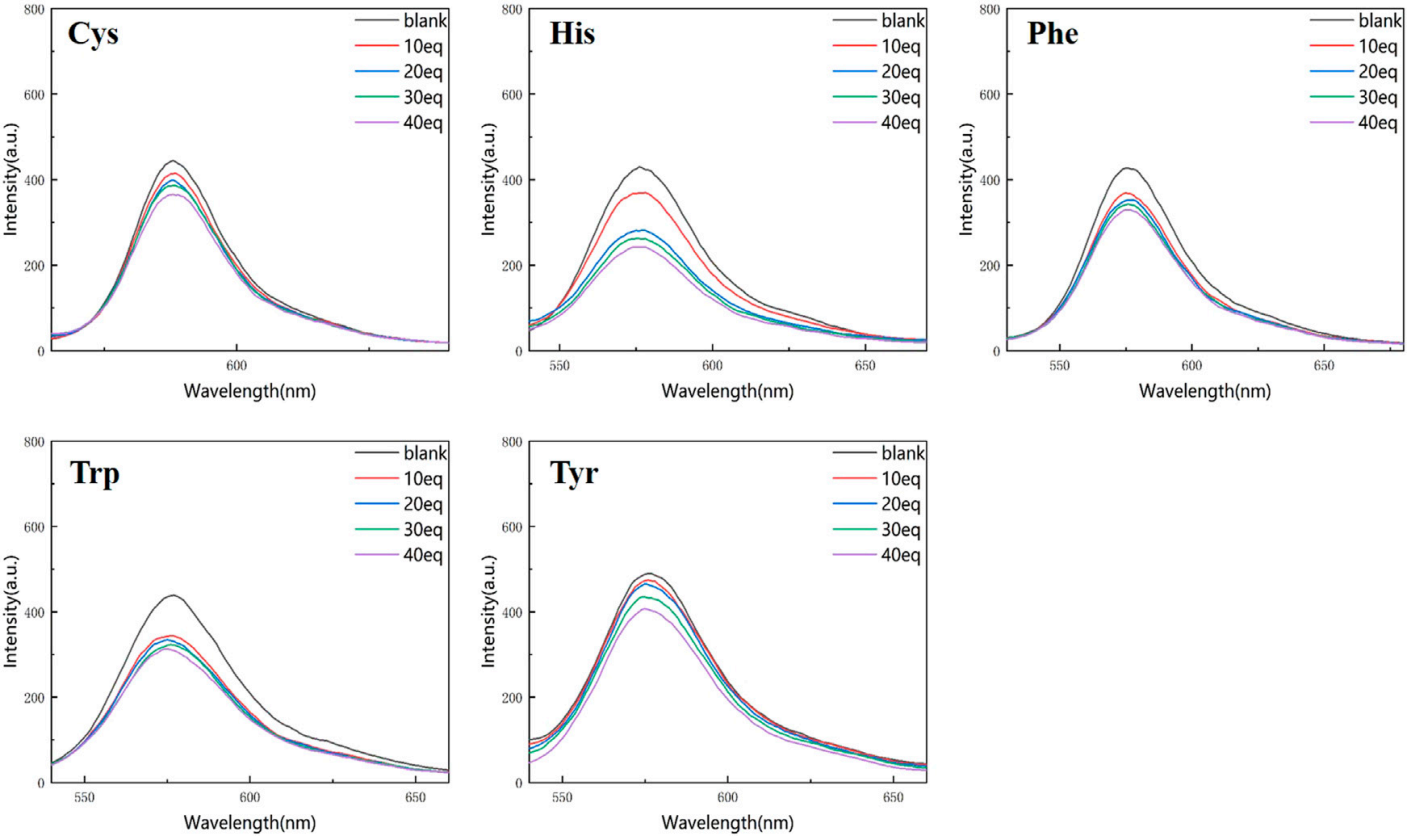
Fluorescence spectrum of RBTPA probe at different concentrations and different amino acids (pH = 4).

Figures 4, 5 show that the total fluorescence spectrum’s wavelength range remains largely constant and that the outcomes are comparable. The intensity of the fluorescence emission is clearly visible at pH = 3, and it is essentially between 1,150 and 1,750, with histamine having the lowest intensity. The intensity of the fluorescence emission is essentially between 400 and 500 at pH = 4. The best effect occurs at pH = 3, and the emission intensity is roughly three times different from the former, which is compatible with the aforementioned UV spectrum data (Figures 4, 5).

Compared with the above four figures, there are obvious curve changes in Figures 2, 3. Each of the corresponding amino acids showed strong reactivity with RBTPA, indicating a response effect between them. Although the fluctuation decline rate of the overall curve is somewhat obvious, there are differences in the absorption intensity. In particular, it shows strong responsiveness at pH = 3 (Table 2).

**TABLE 2:** PL intensity properties of RBTPA in different amino acids.

PL intensity (pH)	Different amino acids				
λ_abs max_, pH = 3	Cys	His	Phe	Trp	Try
	581 nm	576 nm	570 nm	571 nm	570 nm
λ_abs max_, pH = 4	Cys	His	Phe	Trp	Try
	580 nm	573 nm	568 nm	569 nm	570 nm

**FIGURE 6:**
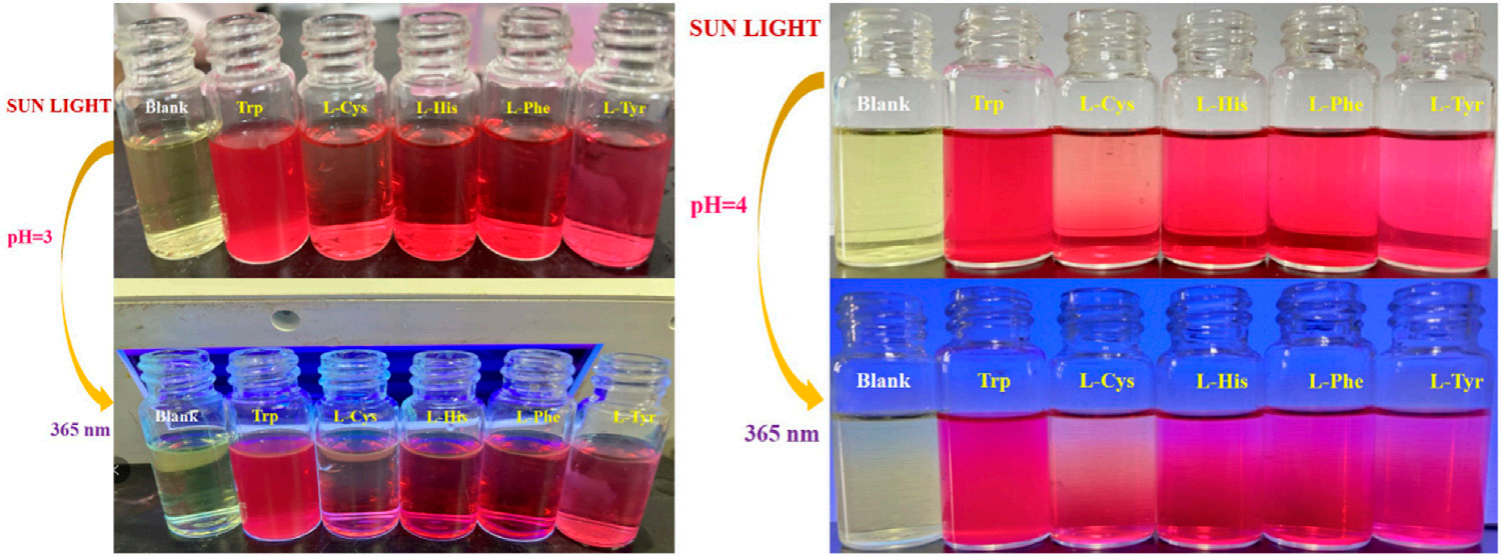
The color response of RBTPA to different amino acids under sunlight and 365 nm irradiation changes.

By comparing the color response recognition of five different amino acids and RBTPA, it can be observed that at pH = 3, the color of RBTPA is opaque except for tryptophan at daylight and 365 nm, and the others are transparent pink or dark red. The color of the blank sample is bright yellow. The results showed that the double response with imino and aldehyde group of RBTPA resulted in color change under the strong acid environment of pH = 3. On the other hand, at pH = 4, the color response recognition of five different amino acids and RBTPA is somewhat dim compared to the former, and there is almost no bright luster at daylight and 365 nm. The results show that one part of RBTPA reacts with imino and aldehyde group in double response, while the other part directly generates acid salt, which affects the fluorescence intensity. These phenomena are in complete agreement with the ultraviolet absorption and fluorescence emission intensity data explained above (Figure 6).

**FIGURE 7:**
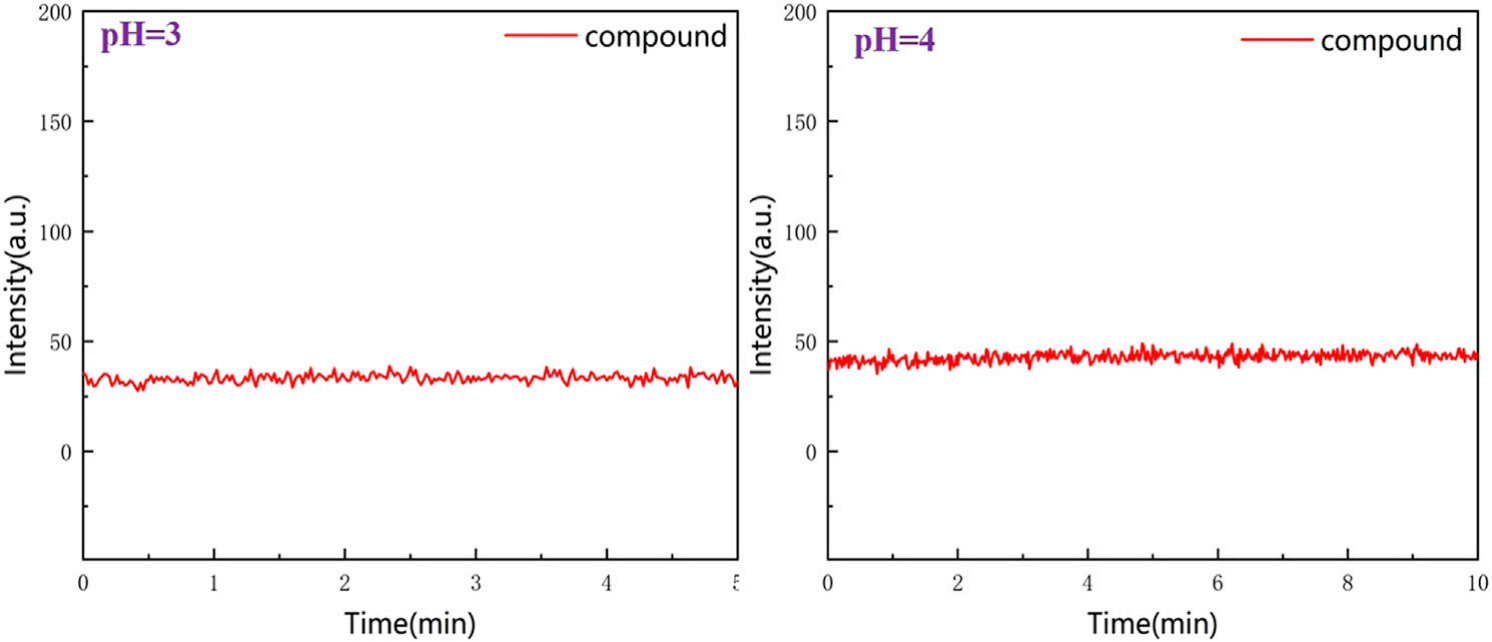
Stability of RBTPA at pH = 3 and 4 acidic conditions.

To observe the stability of RBTPA, a test was performed for 5–10 min at pH = 3 and 4 strong acids. It can be seen from Figure 7 that the two lines maintain a relatively flat state, indicating that the compound RBTPA does not change under the condition of strong acid, and is a stable fluorescent probe (Figure 7).

**FIGURE 8:**
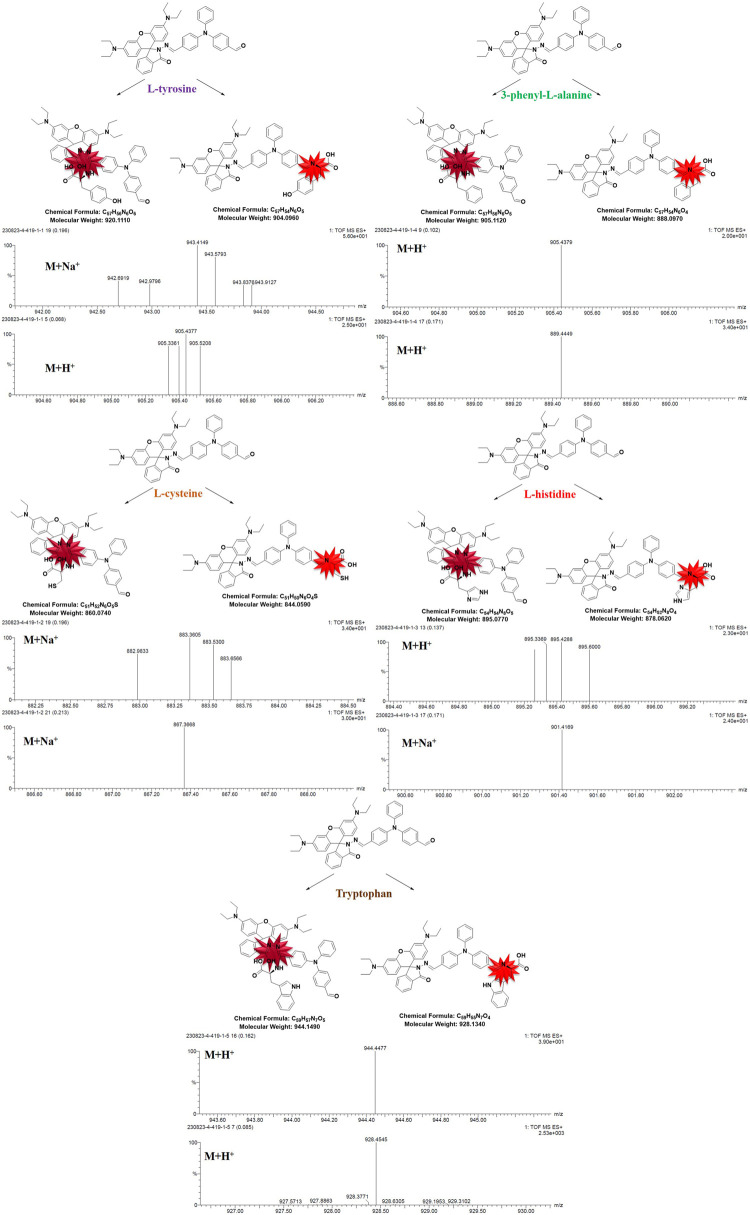
Qualitative analysis of RBTPA combined with five amino acids by mass spectrometry.

In order to further determine the binding of RBTPA to different amino acids in acidic environments for qualitative analysis, each response reaction solution was tested in detail by mass spectrometry. It can be seen from Figure 8 that the mass spectra of tyrosine and phenylalanine are 943.41, 905.43, 905.43, and 889.44, respectively. It can be analyzed as (M + Na^+^), (M + H^+^), (M + H^+^), and (M + H^+^) respectively. By analogy method, cysteine and can be resolved as respectively histidine (M + Na^+^), (M + Na^+^), (M + H^+^) and (M + Na^+^); tryptophan can be resolved as (M + H^+^) and (M + H^+^).

## Conclusion

A novel RBTPA fluorescent probe for rapid and efficient identification of different amino acids is described in this paper. It has the characteristics of simple preparation, strong practicability and short reaction time. Among them, the high efficiency fluorescence spectrometry-mass spectrometry is the highlight of this project. The response of different amino acids can be preliminarily determined by the change of fluorescence chromophores, and then qualitative analysis of their molecular weight can be determined by mass spectrometry. The probe is suitable for research or diagnosis in the field of disease easily and quickly. It will provide important reference for developing more fluorescent probes in the same field in the future.

## Data Availability

The original contributions presented in the study are included in the article/supplementary material, further inquiries can be directed to the corresponding authors.
